# *Propionibacterium acnes* Osteomyelitis after Intraosseous Cannulation in a Child

**DOI:** 10.1155/2019/7170154

**Published:** 2019-12-06

**Authors:** Keegan A. Cole, Samik Banerjee, John A. Dipreta

**Affiliations:** Department of Orthopaedic Surgery, Albany Medical Center, Albany, New York 12208, USA

## Abstract

*Propionibacterium acnes* osteomyelitis secondary to intraosseous (IO) cannulation is not well documented in literature. We report here an extremely rare incident of *P. acnes* tibial osteomyelitis at the IO access site, in a 4-year-old child, who presented 3 months after an episode of fluid resuscitation for streptococcal toxic shock syndrome necessitating irrigation and debridement and prolonged antibiotic therapy. We advocate for heightened awareness of osteomyelitis in patients with continued pain after IO cannulation. Low-grade persistent symptoms may be caused by less virulent organisms and may dictate need for early magnetic resonance imaging studies for diagnosis and treatment planning.

## 1. Introduction

Intraosseous (IO) cannulation was first described in the 1920s and is a widely accepted method of vascular access when peripheral venous access cannot be rapidly obtained [1]. Complications related to device insertion are rare, including compartment syndrome, fracture, air embolism, fat embolism, skin necrosis, and osteomyelitis [2]. Osteomyelitis is a particularly rare complication occurring in 0.6% of 4,720 pediatric IO cannulations [3]. Most common organisms responsible for osteomyelitis include *Staphylococcus aureus*, coagulase-negative staphylococci, *Kingella kingae*, and gram-negative bacilli [4].

*Propionibacterium acnes* (*P. acnes*, a.k.a. *Cutibacterium acnes*) infections are of recent interest as causative bacteria in prosthetic joint infections particularly of the shoulder. Eradication of *P. acnes* with preprocedural skin decontamination is difficult as the bacterium resides deep within confines of the pilosebaceous glands [5–8]. Tibial osteomyelitis from *P. acnes*, however, is extremely rare with limited case reports in the literature and, to the best of our knowledge, has not been previously reported after IO device insertion [9]. *P. acnes* osteomyelitis diagnosis is difficult to establish due to its fastidious nature and slow *in vitro* growth in culture media. We report a case of a 4-year-old patient with *P. acnes* tibial osteomyelitis secondary to IO cannulation during treatment for streptococcal toxic shock syndrome.

## 2. Case Report

A 4-year-old male presented to the ED complaining of right leg pain and antalgic gait for a three-month duration. Plain radiographs revealed irregular lytic lesions of the distal femur and proximal tibia concerning for osteomyelitis prompting orthopaedic surgery consultation.

**Figure 1:**
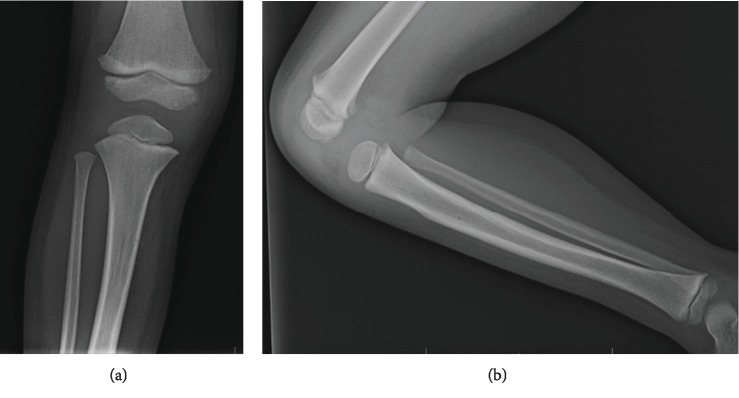
Anteroposterior (a) and lateral (b) plain radiographs of the patient at the time of discharge from initial hospitalization reveal no abnormalities aside from the small cortical defect made by the IO cannula in the proximal tibia.

Three months prior, the child was admitted to the PICU for blood culture-positive streptococcal sepsis with toxic shock syndrome and multiorgan dysfunction. Upon arrival, the child required intubation for ARDS, vasopressin and epinephrine for hypotension, broad-spectrum antibiotics and antivirals, platelets and fresh-frozen plasma for disseminated intravascular coagulation, and stress dose hydrocortisone. Bilateral proximal posteromedial tibia IO catheters were placed for rapid fluid resuscitation and subsequently removed within twenty-four hours after femoral central venous access. After five days in PICU and six days on the pediatric ward, the clinical condition improved, and the child was discharged home with fourteen days of intravenous (IV) ceftriaxone followed by fourteen days of oral amoxicillin for a thirty-nine-day antibiotic course. Prior to discharge, the child was noted to have a right-sided limp. Radiographs obtained revealed no fracture or abnormality aside from the cortical defects from the IO insertion (Figure 1).

**Figure 2:**
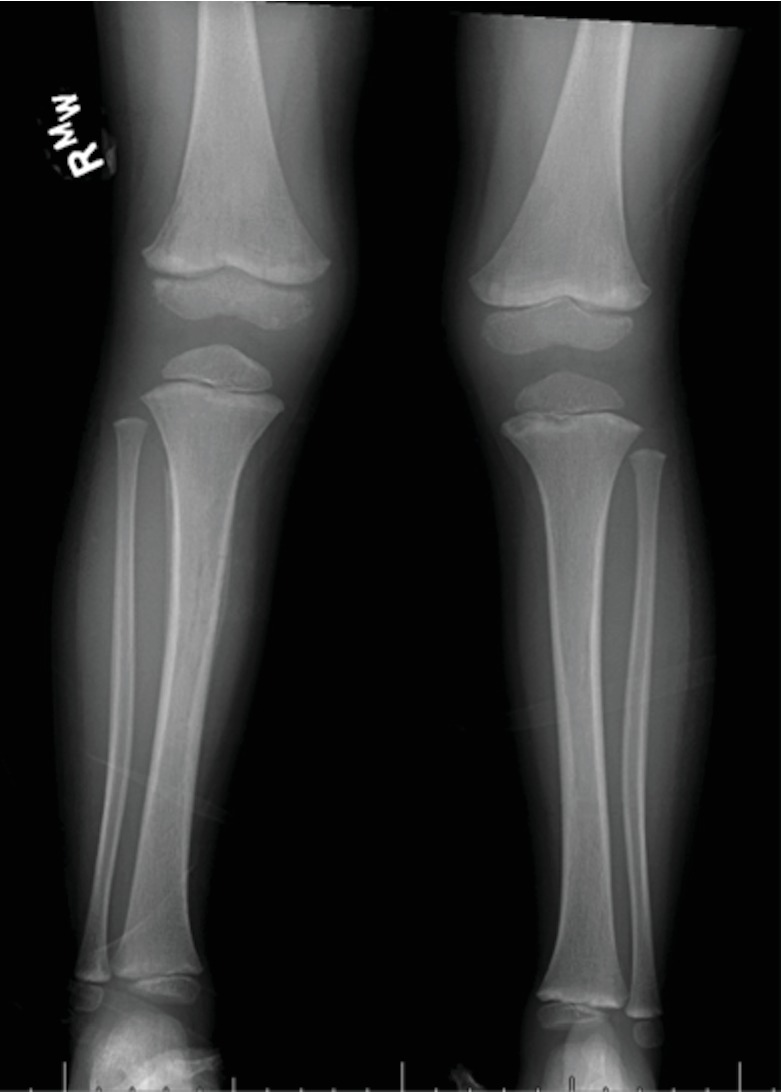
Bilateral anteroposterior plain radiograph of the lower extremities one week after initial discharge demonstrating periosteal reaction of the tibia and striated lucencies of the distal femur.

Eleven days after discharge, the child was readmitted following an outpatient infectious disease specialist consultation revealing persistently elevated inflammatory markers and continued refusal to bear weight. White blood cell (WBC) count was 20,000/*μ*L, erythrocyte sedimentation rate (ESR) of 115 millimeters/hour, and C-reactive protein (CRP) of 12.4 milligrams/liter. Physical exam demonstrated no skin abnormalities, painless hip and knee range of motion, and minimal pain with crawling. Plain radiographs revealed benign periosteal reaction at the IO site and subtle striated lucencies of the right distal femur attributed to disuse osteopenia by a pediatric musculoskeletal radiologist (Figure 2). Ultrasound of the hip and knee revealed no effusions. Pain improved with anti-inflammatories and inflammatory markers normalized, so the child was again discharged home.

**Figure 3:**
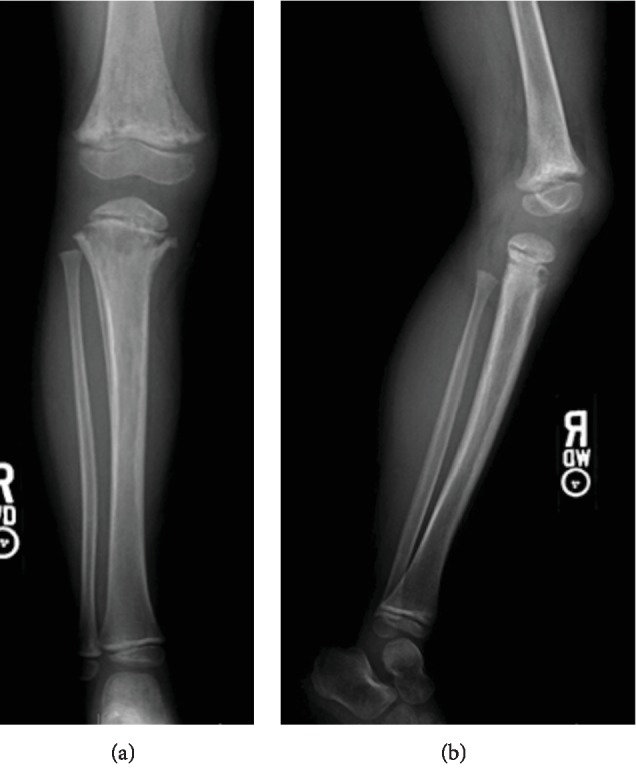
Anteroposterior (a) and lateral (b) plain radiographs of the patient two months after IO cannulation revealing progression of lytic processes in the distal femur and proximal tibia concerning for osteomyelitis.

Two months later during routine follow-up with an ID specialist, radiographs obtained were concerning for osteomyelitis (Figure 3), so the child was sent back to the ED. Orthopaedic surgery was consulted, for the first time, upon arrival. Since the last admission, the child had been limping and complained of pain occasionally but was otherwise happy and playful. Physical exam of the right lower extremity revealed no skin erythema or warmth, full and painless active range of motion of the hip and knee, no tenderness to palpation throughout, and the ability to run and jump with an antalgic gait. There were no documented fevers, and laboratory values showed a WBC count of 8,100/*μ*L, ESR of 10 millimeters/hour, and CRP of 0.2 milligrams/liter. Plain radiographs demonstrated progression of an osteolytic process involving the distal femur and proximal tibia with increased tibial periosteal reaction (Figure 3).

**Figure 4:**
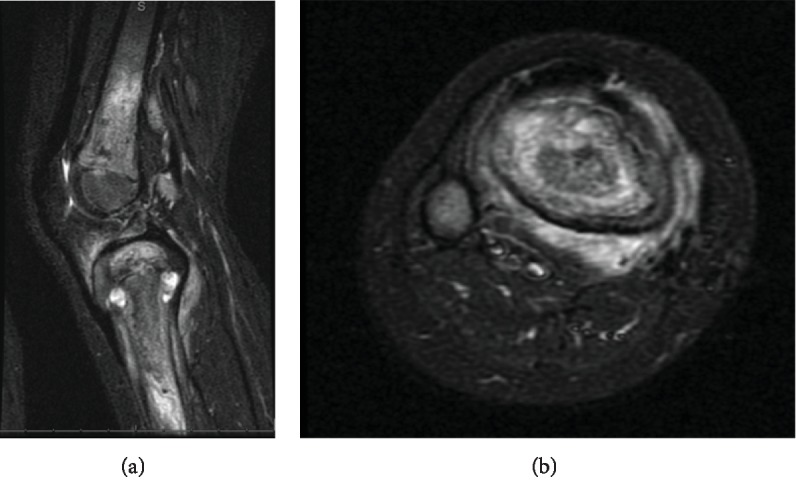
Sagittal (a) and axial (b) short tau inverted recovery (STIR) magnetic resonance imaging demonstrating extensive femoral and tibial edema consistent with osteomyelitis. In addition, two tibial abscesses distal to the physis and posteromedial muscle edema concerning for myositis were noted.

Given the history of tibial IO access during prior hospitalization combined with radiographic findings, the clinical concern for osteomyelitis was high despite normal inflammatory markers. Magnetic resonance imaging (MRI) was chosen to narrow the diagnosis, evaluate the extent of pathology, and determine treatment. MRI revealed distal femur and proximal tibia osteomyelitis with intraosseous abscesses and necrotic bone in the tibia (Figure 4). There was also periosteal reaction with adjacent edema on the posteromedial tibia.

After multidisciplinary discussion with pediatrics and infectious disease specialists, open irrigation and debridement by orthopaedic surgery was chosen over interventional radiology biopsy given the possibility of intra- and extraosseous abscesses. A posteromedial incision over the tibia was made under fluoroscopic guidance targeting the most enhancing area on MRI while simultaneously avoiding the physis. Some thickening and inflammation of the tissues were noted; however, there was no gross purulence or fluid collections. Aspiration attempts from the posterior extraosseous fluid collections were unsuccessful and were followed by posterior dissection revealing normal appearing tissues. The tibial periosteum was elevated, revealing normal appearing bone, and a cortical window was then made to evaluate for the presence of intramedullary purulence. However, no metaphyseal abscesses or purulence was found. Metaphyseal bone was curetted and 3 sets of soft issue and bone samples from the inflamed area were sent for culture and sensitivity testing and histopathological examination. Since the cross-sectional imaging findings in the femur were minor in comparison to the tibia and exploration of the tibia was not impressive, it was decided to avoid surgery on the femur to reduce the morbidity of the procedure. The wound was copiously irrigated and closed over a drain.

After six days, one metaphyseal bone culture identified *Propionibacterium acnes*. Histopathology from the bone demonstrated chronic inflammatory changes. Infectious disease was consulted, and the child was placed on three weeks of intravenous ceftriaxone to be followed by three additional weeks of oral antibiotics. At the most recent three-month follow-up, the child has been pain free and bearing full weight on the affected extremity.

## 3. Discussion

*P. acnes* are gram-positive anaerobic-aerotolerant skin commensal bacilli that are almost ubiquitously present in pilosebaceous-rich areas of the skin. Tibial osteomyelitis caused by *P. acnes* is extremely rare, with isolated case reports published in the literature [9]. Recently, there has been increasing interest in *P. acnes-*related infections due to its causative role in prosthetic joint infections in upper and lower extremities and the difficulties in eradicating the organism despite skin decontamination as these bacilli reside deep within the pilosebaceous glands [5–8]. Intraosseous device insertion-related infections are rare events with scattered reports describing *Staphylococcus aureus* osteomyelitis occurring after rapid intraosseous vascular access [2, 10]. However, to the best of our knowledge, tibial *Propionibacterium acnes* osteomyelitis in a child after intraosseous cannulation for fluid resuscitation, as demonstrated in our patient, has not been previously reported.

Due to indolence and chronicity of symptoms, lack of systemic signs, dearth of physical examination findings, unremarkable laboratory values, and low virulence, there is often delay in diagnosing musculoskeletal infections caused by these opportunistic bacilli [11]. Moreover, the absence of intraoperative purulence, and near-normal osseous and soft-tissue structures on exploration, can be confusing with regard to the appropriateness of tissue sampling for diagnosis. Bone and soft-tissue specimens for culture and sensitivities along with bone biopsy for pathological examinations may be necessary for diagnosing these infections. Additionally, due to the fastidious nature of the organism, it may take anywhere from 5 to 30 days for microbiological identification [12]. Recent techniques using PCR may provide faster, easier, and more accurate methods for diagnosis [13].

As these bacilli form part of the normal skin, conjunctival, external ear canal, and large intestinal flora, contamination of specimens is not uncommon. Asseray et al., in their study of 65 patients with a variety of infections caused *P. acnes*, found the diagnostic accuracy of more than 90% in the presence of two or more positive deep cultures along with either previous surgery, local signs of infection, necrosis, orthopaedic hardware, or inflammatory biological parameters to distinguish infection over contamination [14]. The authors also found that the diagnostic probability was more than 90% in the presence of one positive deep culture with 3 additional minor criteria including perioperative findings of infection, necrosis, hardware loosening, and ≥2 prior surgical procedures. Some authors have reported that absence of local sepsis with only one-tissue positive culture may not preclude infection with *P. acnes*-related prosthetic joint infections [15, 16]. It is particularly difficult to make a suitable decision whether antibiotic therapy is justified in these scenarios [15, 16]. Dramis et al., in their study of 56 patients with *P. acnes* prosthetic joint infections, found that 7 of these patients had one-tissue culture-positive infection during revision surgery for aseptic loosening that was then treated with a 6-week course of antibiotic therapy with no recurrence of infection at 1- to 5-year follow-up [16]. Thirty-eight patients in their series had one-tissue culture-positive *P. acnes* infection that was not treated with antibiotic therapy and had no recurrence of infection at final follow-up. [16]

Although debatable, we are inclined to believe that in the presence of clinical symptoms, hyperintense STIR MRI lesions at the previous intraosseous cannulation sites and one positive culture may provide sufficient evidence to support diagnosis of propionibacterium osteomyelitis and warrant antibiotic therapy to determine resolution of symptoms. Similar to earlier reports documenting abnormal levels of inflammatory markers in less than 20% patients with *P. acnes* infections, we did not find any local evidence of tissue necrosis or preoperative abnormal laboratory inflammatory markers such as elevated ESR and CRP [17].

In conclusion, *P. acnes* osteomyelitis is an unusual diagnosis after any percutaneous procedure in the absence of hardware. These infections are difficult to diagnose and may need early cross-sectional imaging in the presence of atypical symptoms and absence of radiographic or physical findings. Differentiating infection from contamination can be challenging. Symptomatic lesions found on MRI in the presence of one positive culture may necessitate antibiotic therapy for treating *P. acnes* osteomyelitis.

## References

[1] Foex B. A. (2000). Discovery of the intraosseous route for fluid administration. *Journal of Accident & Emergency Medicine*.

[2] Barlow B., Kuhn K. (2014). Orthopedic management of complications of using intraosseous catheters. *American Journal of Orthopedics*.

[3] Rosetti V. A., Thompson B. M., Miller J., Mateer J. R., Aprahamian C. (1985). Intraosseous infusion: an alternative route of pediatric intravascular access. *Annals of Emergency Medicine*.

[4] Lew D. P., Waldvogel F. A. (2004). Osteomyelitis. *The Lancet*.

[5] Nodzo S. R., Westrich G. H., Henry M. W., Miller A. O. (2016). Clinical analysis of *Propionibacterium acnes* infection after total knee arthroplasty. *The Journal of Arthroplasty*.

[6] Frangiamore S. J., Saleh A., Grosso M. J. (2015). Early versus late culture growth of *Propionibacterium acnes* in revision shoulder arthroplasty. *The Journal of Bone and Joint Surgery*.

[7] Bacle G., Sikora S. K., Ek E. T. H. (2017). *Propionibacterium acnes* infection of a metacarpophalangeal joint arthroplasty. *The Journal of Hand Surgery*.

[8] Saltzman M. D., Nuber G. W., Gryzlo S. M., Marecek G. S., Koh J. L. (2009). Efficacy of surgical preparation solutions in shoulder surgery. *The Journal of Bone and Joint Surgery*.

[9] Bachmeyer C., Blanchard M., Zeller V. (2010). Tibial chronic osteomyelitis due to *Propionibacterium acnes* in a patient with sickle cell anemia. *International Journal of Infectious Diseases*.

[10] Yee D., Deolankar R., Marcantoni J., Liang S. Y. (2017). Tibial osteomyelitis following prehospital intraosseous access. *Clinical Practice and Cases in Emergency Medicine*.

[11] Shields M. V., Abdullah L., Namdari S. (2016). The challenge of *Propionibacterium acnes* and revision shoulder arthroplasty: a review of current diagnostic options. *Journal of Shoulder and Elbow Surgery*.

[12] Wang B., Toye B., Desjardins M., Lapner P., Lee C. (2013). A 7-year retrospective review from 2005 to 2011 of *Propionibacterium acnes* shoulder infections in Ottawa, Ontario, Canada. *Diagnostic Microbiology and Infectious Disease*.

[13] Nakamura M., Kametani I., Higaki S., Yamagishi T. (2003). Identification of *Propionibacterium acnes* by polymerase chain reaction for amplification of 16S ribosomal RNA and lipase genes. *Anaerobe*.

[14] Asseray N., Papin C., Touchais S. (2010). Improving diagnostic criteria for Propionibacterium acnes osteomyelitis: a retrospective analysis. *Scandinavian Journal of Infectious Diseases*.

[15] Lutz M. F., Berthelot P., Fresard A. (2005). Arthroplastic and osteosynthetic infections due to *Propionibacterium acnes*: a retrospective study of 52 cases, 1995-2002. *European Journal of Clinical Microbiology & Infectious Diseases*.

[16] Dramis A., Aldlyami E., Grimer R. J., Dunlop D. J., O’Connell N., Elliott T. (2009). What is the significance of a positive *Propionibacterium acnes* culture around a joint replacement?. *International Orthopaedics*.

[17] Pottinger P., Butler-Wu S., Neradilek M. B. (2012). Prognostic factors for bacterial cultures positive for Propionibacterium acnes and other organisms in a large series of revision shoulder arthroplasties performed for stiffness, pain, or loosening. *The Journal of Bone and Joint Surgery*.

